# Population pharmacokinetics of remifentanil in infants and children undergoing cardiac surgery

**DOI:** 10.1186/1471-2253-9-5

**Published:** 2009-07-27

**Authors:** Wai Johnn Sam, Gregory B Hammer, David R Drover

**Affiliations:** 1 Department of Anesthesia, Stanford University, Stanford, California USA; 2 Department of Anesthesia and Pediatrics, Stanford University, Stanford, California, USA

## Abstract

**Background:**

The aim of this study was to provide a model-based analysis of the pharmacokinetics of remifentanil in infants and children undergoing cardiac surgery with cardiopulmonary bypass (CPB).

**Methods:**

We studied nine patients aged 0.5 to 4 years who received a continuous remifentanil infusion via a computer-controlled infusion pump during cardiac surgery with mildly hypothermic CPB were studied. Arterial blood samples taken prior to, during and after CPB were analyzed for remifentanil concentrations using a validated gas-chromatographic mass-spectrophotometric assay. We used population mixed-effects modeling to characterize remifentanil pharmacokinetics. The final model was evaluated by its predictive performance.

**Results:**

The pharmacokinetics of remifentanil was described by a 1-compartment model with adjustments for CPB. Population mean parameter estimates were 1.41 L for volume of distribution (V) and 0.244 L/min for clearance. V was increased during CPB and post-CPB to 2.41 times the pre-CPB value. The median prediction error and the median of individual median absolute prediction error were 2.44% and 21.6%, respectively.

**Conclusion:**

Remifentanil dosage adjustments are required during and after CPB due to marked changes in the V of the drug. Simulations indicate that a targeted blood concentration of 14 ng/mL is achieved and maintained in 50% of typical patients by administration of an initial dose of 18 μg remifentanil followed by an infusion of 3.7 μg/min before, during and post-CPB, supplemented with a bolus dose of 25 μg given at the start of CPB.

## Background

Remifentanil is a selective μ-opioid receptor agonist that produces intense analgesia of rapid onset and ultra short duration [1]. Accordingly, remifentanil provides the benefits of a high-dose opioid anesthetic during cardiac surgery (e.g. minimal sympathetic response to tracheal intubation and sternotomy) while allowing tracheal extubation in the operating room immediately following surgery.

CPB may alter the pharmacokinetics of anesthetic agents due to hypothermia, hemodilution, exclusion of the lungs from the circulation and a decrease in plasma protein concentration [2]. However, the impact of CPB on the pharmacokinetics of remifentanil in infants and children has not been fully investigated. Davis et al [3] studied the pharmacokinetics of remifentanil before and after CPB in 12 pediatric patients undergoing repair of an atrial septal defect. They found that after CPB clearance values increased by 20% (from 38.7 ± 9.6 to 46.8 ± 14 mL/min/kg), without a meaningful change in the coefficient of variation. The authors concluded that the pharmacokinetics of remifentanil is predictable following CPB. However, this study failed to investigate the pharmacokinetics of a remifentanil infusion during CPB.

An unanticipated decrease in remifentanil plasma concentration during CPB may be detrimental. Significant intra-operative stress responses, postoperative complications and increased mortality have been reported in infants receiving inadequate analgesia during cardiac surgery [4,5]. Therefore, the aim of our investigation was to determine the effect of CPB on the pharmacokinetics of remifentanil in infants and children during all phases of cardiac surgery using mixed-effect modeling in order to inform accurate dosage titration.

## Methods

Nine infants and children scheduled for open-heart surgery requiring CBP were studied after Institutional Review Board approval and written informed parental consent were obtained. These patients were part of a prospective, randomized, controlled clinical trial to define the opioid analgesic requirement after a remifentanil-based anesthetic with or without spinal anesthetic blockade (SAB). Inclusion criteria were age 3 months to 6 years and planned tracheal extubation in the operating room after surgery (e.g. absence of severe pulmonary hypertension or heart failure). Exclusion criteria were contraindication to SAB and failure to obtain informed consent. Details of the study have been described elsewhere [6].

Premedication was given to patients over the age of 1 year (midazolam 0.5–0.75 mg/kg by mouth). Following placement of standard monitors, anesthesia was induced with sevoflurane and tracheal intubation was performed after administration of rocuronium. Anesthesia was maintained with isoflurane 0.3% and a continuous infusion of remifentanil with or without SAB with tetracaine (0.5 – 2.0 mg/kg) and morphine (0.007 mg/kg). Remifentanil was infused with a target-controlled infusion system programmed with parameters obtained from Minto et al [7] to maintain a constant predicted blood target concentration of 1–8 ng/mL. Remifentanil infusion rates were adjusted according to the judgment of the investigator in order to achieve a target mean arterial blood pressure (i.e. 50 to 60 mm Hg). Arterial blood pressure, heart rate, oxygen saturation and dosing history of remifentanil were continuously recorded during the surgery. At the completion of surgery, tracheal extubation was performed in the operating room and supplemental analgesia was achieved with fentanyl 0.003 mg/kg IV every 10 minutes as needed.

Prior to CPB, anticoagulation was established with an initial bovine heparin dose of 400 U/kg and additional heparin was administered during CPB to maintain celite activated clotting time (ACT) greater than 480 seconds. Non-pulsatile CPB was performed with a hollow fiber membrane oxygenator (Terumo CapioxC RX05, Terumo Cardiovascular Systems, Ann Arbor, MI), uncoated polyvinyl chloride bypass tubing and cannulae, and non-occlusive roller pump. The circuit was primed with normal saline, 25% albumin, mannitol, sodium bicarbonate, calcium chloride, methylprednisolone (30 mg/kg) and heparin. CPB circuit volumes were 450 mL for patients < 10 kg, 800 mL for patients 10–15 kg and 1,000–1,200 mL for patients > 15 kg. Banked packed red blood cells and fresh frozen plasma were added to achieve a hematocrit of about 30% during initiation of CPB. CPB flow rates were 200 mL/kg for infants with body weight less than 5 kilograms (kg), 150 mL/kg for those between 5 and 9 kg and 125 mL/kg for those between 10 and 17 kg; flows of 2.4 L/m^2 ^were used in children over 17 kg. An initial dose of cardioplegia of 30 mL/kg was given, followed by 10 mL every 10–20 minutes thereafter. Hypothermia (28 – 32°C) was induced in all patients and blood gases were regulated according to alpha-stat regimen. Myocardial preservation was achieved using cold crystalloid cardioplegia. Target post-CPB hematocrit values varied from 35% to 50% depending upon the patient's cardiac and respiratory status. Antifibrinolytic agents were not administered.

Conventional ultra-filtration (CUF) was performed throughout CPB to achieve a filtrate volume of at least 120 mL/kg. Fluids (crystalloid, red blood cells or fresh frozen plasma) were added when necessary to provide sufficient volume in the CPB circuit to permit ultrafiltration. The polysulfone hemofilter used (MinntechC HPH 400; Minntech Corporation, Minneapolis, MN) employs hollow fiber technology and is rated to have a filtration cut-off to particles greater than 65,000 Daltons (Da). A transmembrane pressure gradient of at least 200 mm Hg was applied during ultrafiltration. After the addition of blood products, hemofiltration of the CPB circuit prime was performed before CPB to adjust pH and electrolyte concentrations and to remove inflammatory mediators. Filtrate volume from pre-CPB filtration ranged from 100 mL to 200 mL.

Arterio-venous modified ultra-filtration (MUF) was initiated on selected patients after separation from CPB according to the surgeons' preferences. Blood from the aortic cannula and from the CPB circuit venous reservoir was pumped through the hemofilter and was then warmed by a coiled heat exchanger (Medtronic MYOtherm XPR cardioplegia delivery system, Medtronic Inc., Minneapolis, MN) and returned via the cardioplegia circuit to the venous cannulae. Infusion rates were adjusted to maintain appropriate central venous and/or left atrial pressures. MUF was terminated when red cell salvage of circuit contents was judged by the perfusionist to be complete.

Whole blood arterial samples for assay of remifentanil concentrations were obtained before and 5 min after each adjustment of the infusion rate. No samples were taken prior to the initiation of remifentanil infusion. Samples were initially aspirated into heparinized syringes, immediately transferred to tubes containing 50% citric acid to inactivate plasma esterases and kept frozen at -20°C. Remifentanil concentrations were determined using a validated gas-chromatographic mass-spectrophotometric (GC-MS) assay with inter-, intraassay coefficients of variation and lower limit of quantification of 4.6%, 4.0% and 0.5 ng/mL, respectively [3].

Pharmacokinetic data were analyzed via a population approach implemented using the NONMEM V program (Globomax LLC, Hanover, MD) with PREDPP subroutines ADVAN1 TRANS2 [8]. First-order conditional estimation with interaction was used for the estimation of model parameters. One- and two- compartmental models were evaluated as the structural pharmacokinetic models. For the purpose of this analysis, the study was subdivided into two time intervals: CPB and non-CPB, where CPB was defined as the period between initiation and termination of CPB and non-CPB was defined as both the periods from the start of the remifentanil infusion until CPB was initiated and the periods between termination of CPB and completion of blood sampling. Relationships between the categorical covariates such as CPB, gender, application of SAB, MUF and a pharmacokinetic model parameter p were modeled as shown below:

(1)

(2)

where TV(P) refers to the typical value of the pharmacokinetic parameter for a patient with the reference covariate value and θ_covariate_refers to the estimated fractional change in the typical value of the pharmacokinetic parameter for the investigated covariate.

Allometric scaling was implemented to assess the influence of body size on the pharmacokinetic parameters [9].

(3)

where P_i _is the parameter in the ith individual, W_i _is the weight in the ith individual, and P_std _is the parameter in an individual with a weight W_std _of 70 kg. The PWR exponent was 0.75 for CL and 1 for volume of distribution. The effects of continuous covariates such as age, body weight were assessed for their influence on the pharmacokinetic parameters according to the following equation:

(4)

where TV(P) is the typical value of P for a patient with the mean covariate value and θ_covariate _is the estimated effect for the covariate on P. In addition, the effect of body temperature on CL of remifentanil was modeled both as a linear function and an exponential function:

(5)

(6)

where TVCL is the model predicted value for CL given the value for temperature. θ_CL _represents the population central tendency for CL. θ_CL, TEMP _represents a parameter quantifying the effect of temperature on CL, and TEMP is the body temperature in °C.

Using the basic pharmacokinetic model, each potential covariate was separately incorporated and tested for statistical significance by use of the NONMEM objective function and the standard errors of the parameters. The likelihood ratio test at the significance level α = 0.01 was used to discriminate between alternative hierarchical models. The α level of 0.01 corresponds to a reduction of 6.64 (χ^2^, p < 0.01; 1 degree of freedom) in the minimum objective function when 1 parameter is added to the model and was used to examine significance. In addition to the minimum objective function, diagnostic goodness-of-fit plots were used for model building and selection. If more than one significant covariate was found, the covariate model causing the largest decrease in objective function was chosen as a basis to explore the influence of additional covariates sequentially with the use of the same criteria.

An exponential variance model (Equation 7) was used to describe the inter-individual variability in the pharmacokinetic parameters with the assumption that the parameters are log-normally distributed:

(7)

where p_i _refers to the individual value of the respective pharmacokinetic parameter in the ith individual, θ is the typical value of the parameter, and exp(ρ_i_) expresses the random difference between θ and p_i_. Values of η_i _are assumed to be independently multi-variate and normally distributed, with mean zero and diagonal variance-covariance matrix Ω with diagonal elements (ω_1_^2^,..., ω_m_^2^).

A proportional and combined additive and proportional models for residual variability for pharmacokinetic observations were evaluated (Equations 8 and 9).

(8)

(9)

where C_ij _is the jth remifentanil concentration of the ith individual predicted by the pharmacokinetic model, and Y_ij _is the measured remifentanil concentration. The residual departure of Y_ij _from C_ij _is represented by ε_ij_. Values of ε_ij _are assumed to be independently and normally distributed, with mean zero and variance σ^2^.

The likelihood ratio test at the significance level α = 0.01 was used to discriminate between hierarchical models. This corresponds to a decrease of ≥ 6.635 (one parameter difference) in the minimum objective function (-2 × logarithm of the likelihood of the results), as the difference in objective function between hierarchical models is approximately χ^2^distributed. When more than 1 parameter was added, a decrease in the objective function of ≥ 9.210, 11.345 and 13.277 was needed at the significance level of α = 0.01 for 2, 3 and 4 degrees of freedom, respectively.

Predictive performance of the final pharmacokinetic model was evaluated by examining the prediction error (PE), which is a retrospective analysis of the quality of fit [10]. PE is defined as follows:

(10)

where C_m _is the measured remifentanil concentration and C_p _is the predicted remifentanil concentration. The intra-subject bias (inaccuracy) and precision of the predictions were assessed by quantifying the median prediction error (MDPE) (median of all MDPE_i_) and median absolute weighted residual (MDAPE) (median of all MDAPE_i_), respectively. MDPE for the *i*th patient is given by:

(11)

where N_i _is the number of samples obtained for the *i*th patient and *j *is the sample number for the *i*th patient. MDAPE for the *i*th patient is given by:

(12)

where N_i _is the number of samples obtained for the *i*th patient and *j *is the sample number for the *i*th patient. Model goodness-of-fit was checked by using diagnostic plots such as the PE over time plot and observed versus predicted plot. The final optimal model was selected based on the objective function, the predictive accuracy and goodness-of-fit plots.

The predictive performance of the population pharmacokinetic parameters of remifentanil by Minto et al [7] used to program STANPUMP was assessed by the prediction error (PE) and residual error plot. The intra-subject bias and precision of the predictions were assessed by quantifying the MDPE and MDAPE, respectively. The residual error plot is the percentage ratio of the measured concentration over the predicted concentration versus time.

We assessed the stability and robustness of the final models via bootstrapping [11]. We obtained a new replication of original dataset (a bootstrap sample) by 1000 random draws of individual subject's data (with replacement) from the original dataset and we fitted the final model to each new dataset. We compared the final model parameter estimates to the mean of the parameter estimates obtained from 1000 bootstrap replicates of the final model.

Simulation was also performed using parameters of our final pharmacokinetic model to determine the optimal infusion and loading doses of remifentanil needed during the different intervals of cardiac surgery needed to maintain average concentration of 14 ng/mL. A typical patient of weight 11.4 kg and 60 min of surgery were assumed before and after 60 min of CPB for simulation purposes.

## Results

Table 1 lists patient demographic data. Mean total infusion time was 218 ± 77.9 min (mean ± standard deviation, SD). The mean total dose of 721 ± 362 μg (mean ± SD) remifentanil and mean weight-normalized total dose of 65.0 ± 32.6 μg/kg (mean ± SD) remifentanil were administered. Remifentanil concentrations during pre-CPB, intra-CPB and post-CPB were 13.8 ± 7.80 ng/mL (mean ± SD), 12.7 ± 6.39 ng/mL (mean ± SD) and 11.7 ± 7.03 ng/mL (mean ± SD), respectively. No samples measured were below the lower limit of quantification. The infusion rates required to control MAP during pre-, intra- and post-CPB were 0.264 ± 0.205 μg/min/kg (mean ± SD), 0.339 ± 0.120 μg/min/kg (mean ± SD) and 0.321 ± 0.175 μg/min/kg (mean ± SD), respectively. A total of 105 samples from all 9 patients were available for modeling. A total of 34, 23 and 48 samples were obtained during pre-, intra- and post-CPB periods, respectively. The median number and range (in parentheses) of samples obtained during pre-, intra- and post-CPB were 3 (1–9), 3 (1–5) and 7 (3–8), respectively.

**Table 1:** Patient demographics

	Value
Gender (male/female)	5/4
Age (year)^†^	2.19 (0.5–4.0)
Weight (kg)^†^	11.4 (6.4–14.7)
Anesthetic technique	
SAB+REMI/REMI	3/6
Procedures	
Atrial septal defect repair	6
Glenn shunt	2
Supraventricular aortic stenosis repair	1
Resection of pulmonary valve leaflets	1
Reconstruction of pulmonary valve	1
Reduction plasty of aneurysm of main pulmonary artery	1
Division of ductus arteriosus	1
Modified ultrafiltration	
Yes/no	4/5
Duration of CPB (min)*	75.0 ± 33.3

^† ^Values are mean (range)

*Values are mean ± standard deviation

Abbreviations: SAB = spinal anesthetic blockade, REMI = remifentanil

**Figure 1:**
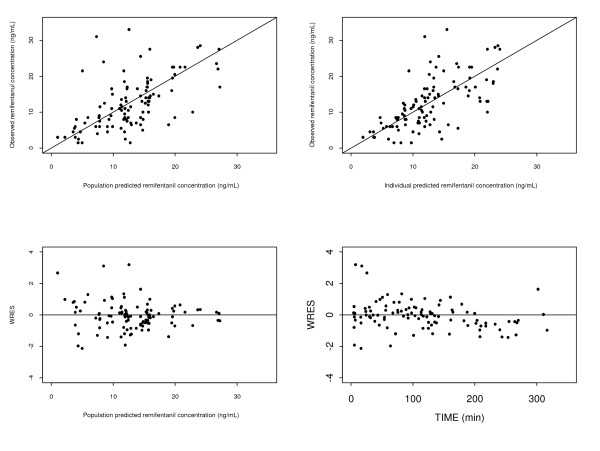
**Basic goodness-of-fit plots for the population pharmacokinetic model**. The line of identity is presented. (Upper left and right panels) Population and individual predicted vs. observed remifentanil concentrations. (Lower left and right panels) Weighted residuals vs. mean predicted and time.

A small decrease in the objective function of 1.33 is associated with the use of a two-compartment model compared with a one-compartment model. Therefore, a one-compartment model was used as the structural model for estimating CPB-adjusted models. A small decrease in the objective function of 0.275 is associated with the use of a combined proportional and additive residual error model compared with a proportional residual error model. Therefore, a proportional residual error model was used. Use of allometric scaling did not result in improvement of the fit as evidenced by a small decrease of 1.162 units in the objective function. The model incorporating changes in V during CPB and post-CPB produced the greatest drop in objective function of 15.928 and was statistically significantly (p < 0.01) better than the one-compartment model. Allowing changes in CL during the CPB did not result in a statistical improvement in fit. Therefore, the V-adjusted one-compartment model is used as the final pharmacokinetic model for remifentanil. The basic goodness-of-fit plots for the population pharmacokinetic model of remifentanil are shown in Figure 1.

**Figure 2:**
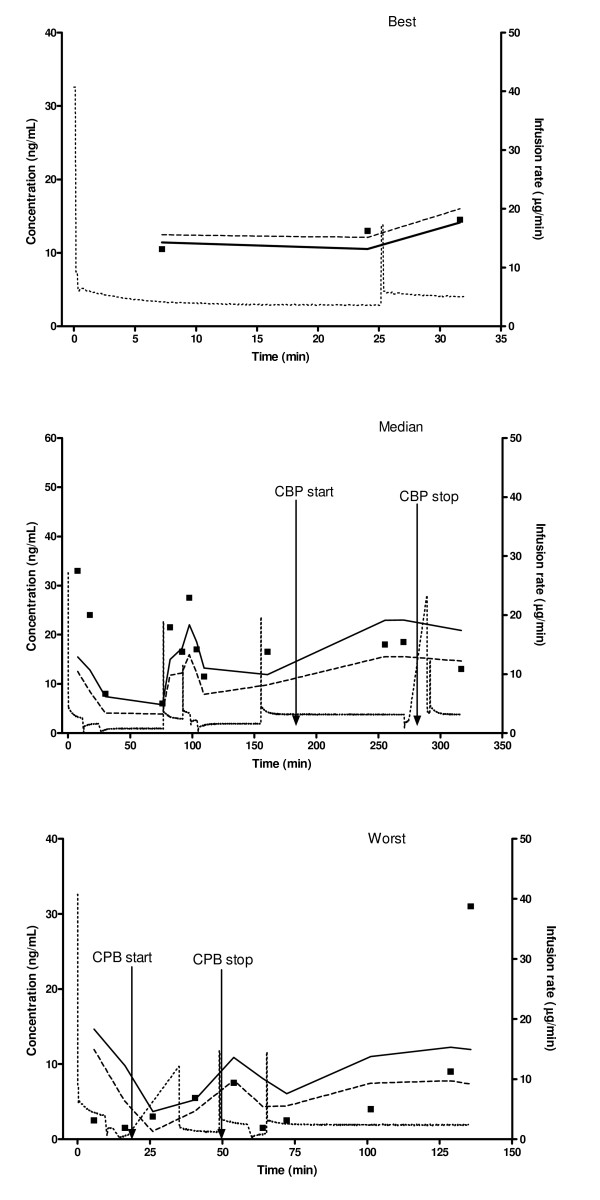
**Best, median and worst pharmacokinetic predictions for remifentanil based on the final pharmacokinetic model (measured concentration (black square), ---- population predicted concentration, —— individual predicted concentration and ... infusion rate)**. Arrows indicate the start and stop times of cardio-pulmonary bypass (CPB).

Table 2 shows the volume and clearance of the final population pharmacokinetic model of remifentanil and the stability of the parameters using the bootstrap resampling procedure. The data supported changing only V with the onset of CPB and post-CPB. With the onset of CPB and post-CPB, V increased 2.41 times of its non-CPB value. The final model estimates were similar to that obtained from 1000 bootstrap replicates of the original dataset, indicating stability of the population pharmacokinetic model. Final weight-normalized population parameter estimates (obtained by normalizing the final population mean estimates with the average weight) are shown in Table 3. For the final pharmacokinetic model, the MDPE for all data points was 2.44% and the MDPE for individual patients ranged from – 45.0% to 14.3%. The plot of PE over time shows random distribution of PEs about the line of zero PE and ranges between -100 to 100% (not shown). MDAPE for the individual patients ranged from 8.11% to 61.2% and the median individual MDAPE is 21.6%. Figure 2 shows the worst, median and best fits (as assessed by the MDAPEs) for the final pharmacokinetic model.

**Table 2:** Population pharmacokinetic parameters and the stability of the parameters using the bootstrap resampling procedure.

	Original data		1000 bootstrap replicates	
	Mean estimate	95% C.I.	Mean estimate	95% C.I.
Structural model				
Volume of distribution during pre-bypass, V (L)	1.41	0.491, 2.33	1.57	0.943, 2.81
Clearance, CL (L/min)	0.244	0.197, 0.291	0.249	0.201, 0.300
Effect of bypass and post-bypass on V_PRE_	2.41	1.60, 3.22	2.26	1.66, 2.96
Inter-individual variability ω CL (%)	33.8	19.7, 43.5	31.3	16.0, 41.1
Residual unexplained variability				
Proportional residual error, coefficient of variation (%)	43.8	25.1, 56.7	42.5	29.0, 57.3

C.I., confidence interval

**Table 3:** Pharmacokinetic parameter estimates of remifentanil in pediatric patients undergoing cardiac surgery.

	This study^a^			Pediatric^b^	Adults^c^	Adults^c^	Pediatric^b^	Adults^d^
	Pre-CPB	CPB	Post-CPB	Pre-CPB		CPB	Post-CPB	
**Volumes (mL/kg)**								
V_c_	124	298	298	72.7	41.8 (23–61)	65.9 (22.6–89.0)	83.5	22.6
V_d_^ss^	124	298	298	235	1006 (245–1767)	344 (246–456)	235	456
								
**Clearances **(mL/min/kg)								
CL	21.4	21.4	21.4	38.7	32.5 (32–33)	31 (25–35)	46.8	33
Q	N.A.	N.A.	N.A.	25.5	29.6 (28.7–30.5)	39.8 (28.7–51.2)	37.0	28.7
								
**Half-lives (min)**								
Distribution	N.A.	N.A.	N.A.	0.73	0.43 (0.25–0.6)	0.55 (0.25–0.8)	0.63	0.25
Elimination	4.02	9.65	9.65	8.2	8.35 (6.4–10.3)	13.0 (7.2–19.8)	6.90	19.8

Abbreviations: CPB = cardiopulmonary bypass; V_c _= central volume of distribution; V_d_^ss ^= volume of distribution at steady-state; CL = clearance; Q = intercompartmental clearance, N.A. = not applicable

^a ^Values are presented as population mean

^b ^Values are presented as mean as reported by Davis et al [3]

^c ^Values are presented as mean (range) values as reported by Michelsen et al and Russel et al [12,13]

^d ^Values are presented as mean as reported by Michelsen et al [12]

**Figure 3:**
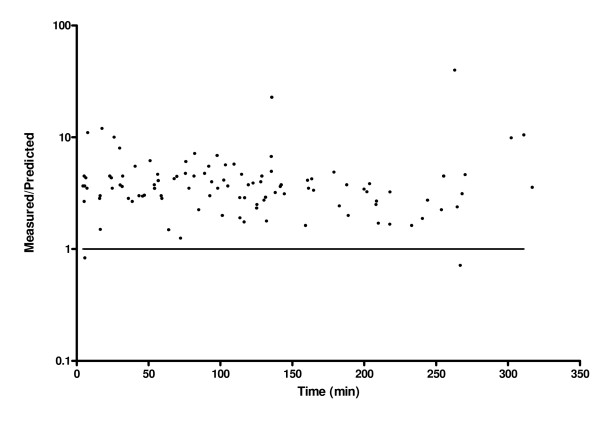
**Residual error plot of measured versus predicted remifentanil concentration using population pharmacokinetic parameters of remifentanil of Minto et al **[7]** for all 9 patients over time**. Shown are the equality line of measured and predicted remifentanil concentration (—) and the residual error (black circles).

The predictive performance of the remifentanil target-infusion system incorporating the population pharmacokinetic parameters for remifentanil of Minto et al [7] showed a poor performance with both MDPE and MDAPE of 256%. The residual error plot showed that the concentrations were consistently under-predicted with time (Figure 3).

**Figure 4:**
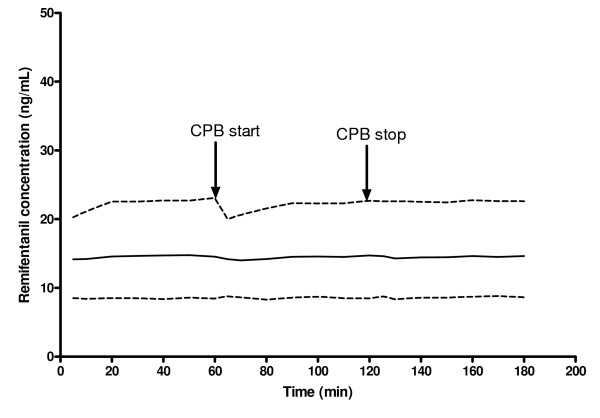
**Simulated pharmacokinetic profile of remifentanil during pre-cardiopulmonary bypass (CPB), intra-CPB and post-CPB for typical patients and receiving an initial loading dose of 18 μg and a 3.7 μg/min continuous infusion of remifentanil before, during and post-CPB with a supplemental bolus dose of 25 μg given at the start of CPB**. The solid line represent the 50^th ^quantile (median) of the simulation, whereas the upper and lower dashed lines represent the 80^th ^and 20^th ^quantiles of the simulation, respectively. Arrows depict the start and stop times of CPB.

Simulation results (Figure 4) showed that an initial loading dose of 18 μg and a 3.7 μg/min continuous infusion of remifentanil before, during and after CPB and a bolus dose of 25 μg given at the start of CPB, achieves and maintains a targeted blood concentration of 14 ng/mL in 50% of the patients. 60% of the simulated patients would achieve blood concentrations between 8.31 to 23.1 ng/mL.

## Discussion

Remifentanil is a widely used analgesic agent during cardiac surgery but little is known about its pharmacokinetics in infants and children undergoing CPB. We found that a 1-compartment pharmacokinetic model adjusted for CPB performed well. The relatively sparse sampling scheme we adopted in this study precludes the development of a multi-compartment model. Although a limited number of blood samples were collected during CPB, use of a V-adjusted CPB pharmacokinetic model produced a statistically significant improvement (p < 0.01) over the basic 1-compartment model.

The results of our study may be compared with those of other investigators studying the pharmacokinetics of remifentanil in pediatric and adult patients undergoing CPB [3,12,13] (Table 3). The population mean volume of distribution at steady-state (Vss) at pre-CPB, 124 mL/kg, estimated by our final CPB-adjusted pharmacokinetic model in this study is smaller than those previously reported in pediatric patients of 235 mL/kg [3,12,13]. However, the population mean V_ss _at CPB and post-CPB, 298 mL/kg is similar to the post-CPB value of 235 mL/kg reported by Davis et al [3]. The relatively small population mean CL estimated in this study (21.4 mL/kg/min) maybe due to differences in patient characteristics from studies previously reported such as cardiac output, which has been reported to be a factor influencing the CL of remifentanil [14]. The elimination half-lives of remifentanil were similar to those determined by previous investigators [3,12,13].

The results of our analysis indicate that V of remifentanil increases by 141% with the institution of CPB and remained increased during post-CPB. This is similar to the findings of Michelsen et al who found that the volume of distribution of remifentanil increased by 86% with institution of CPB, and remained increased during post-CPB [12] in adult patients. Hemodilution due to the CPB prime is the most likely reason for the increase in V. The 141% increase in the V of remifentanil in infants and children during and post- CPB compared to pre-CPB is higher than the expected range as the average CPB prime volume for patients between 10–15 kg is about 800 mL. The discrepancy may be due to drug adsorption to CPB circuit or additional sources of extracellular fluid secondary to rennin and atrial natriuretic peptide release as a result of non-pulsatile renal blood flow during CPB [15]. Our results are also similar to the findings of Davis *et al *[3] who estimated similar volumes of distribution of remifentanil during post-CPB in children undergoing cardiac surgery.

The CL of remifentanil was found to be unaffected by CPB which is similar to that reported by Michelsen et al [12]. Contrary to that reported by Michelsen et al [12] and Russell et al [13], temperature is not found to be a significant factor affecting the CL of remifentanil in this study. This may be due to a limited range of temperature (33.7–38.0°C) and mild hypothermia encountered in the majority of the patients during CPB. Moreover, the relatively short duration of CPB may have hindered the ability to identify changes in CL during bypass. The changes in the elimination half-lives (t_1/2β_) of remifentanil during the CPB and non-CPB follow the same trend and magnitude as the V (Table 3), suggesting that the pharmacokinetic advantage of short recovery from anesthesia post-CPB is modified in pediatric cardiac surgical patients.

MUF, first described by Naik et al [16], reduces total body water, induces hemoconcentration and improves postoperative recovery in children after open heart surgery [16,17]. However, information is lacking regarding how MUF affects the pharmacokinetics of remifentanil in pediatric patients. Our study shows that MUF has little effect on the pharmacokinetics of remifentanil in pediatric cardiac patients.

The population model is able to predict blood concentrations accurately and with good precision as evidenced by a small MDPE and median individual MDAPE of 2.44% and 21.6%, respectively. These, together with the results of the diagnostic plots which showed random uniform scatter around the identity line (Figure 1) and an absence of bias, indicates the suitability of the final pharmacokinetic model as a basis for pharmacokinetic modeling. On the other hand, the poor predictive performance of the remifentanil target-infusion system incorporating the population pharmacokinetic parameters for remifentanil of Minto et al [7] developed using data from adult patients does not meet the criteria for clinical utility [18] and thus has a limited utility in pediatric patients.

One reason for pharmacokinetic analysis is to determine an effective dosing regimen. Therapeutic blood concentrations of remifentanil range from 1.5 ng/mL for sedation to 50 ng/mL for anesthetic effects in both humans and dogs [1,12,19,20]. We selected an average concentration of 14 ng/mL is selected as the therapeutic concentration for the design of an effective dosing regimen for remifentanil. This concentration was chosen as a result of pharmacokinetic-pharmacodynamic analysis of the hypotensive effect of remifentanil in pediatric patients undergoing cranioplasty by the authors, which shows that this concentration leads to a 30% reduction in mean arterial pressure from the baseline. For a typical patient, an initial loading bolus dose of 18 μg remifentanil and a 3.7 μg/min continuous infusion before, during and post-CPB, supplemented with a bolus dose of 25 μg given at the start of CPB, achieves and maintains a targeted blood concentration of 14 ng/mL in 50% of the patients. The supplemental bolus dose is needed to compensate for the increase in V during CPB.

Because the population model was based on pediatric patients with mild hypothermia during CPB, this dosing recommendation may not apply to conditions of moderate to deep hypothermia during CPB as low body temperature has been reported to be a factor reducing CL during CPB [12,13].

One limitation of our pharmacokinetic modeling is our model's failure to account for drug removed from the CPB circuit post-CPB. This problem can be solved by treating the CPB process as a separate compartment from the central compartment and the CPB compartment turn on/off when CPB starts/stops. Unfortunately, the relatively sparse sampling schedule in this study precludes using a multi-compartment model and the use of this approach to correct for the removal of drug from the system after CPB. Thus, we adopted an approach commonly reported in the literature of allowing the pharmacokinetic parameters to change during and after CPB for modeling.

## Conclusion

We developed a CPB-adjusted pharmacokinetic model for remifentanil dosing in infants and children undergoing cardiac surgery with mild hypothermic CPB. Because the V of remifentanil was markedly increased during CPB and remained elevated post-CPB, a supplemental bolus dose of remifentanil is required during CPB in pediatric cardiac anesthesia. Studies are warranted to determine the pharmacokinetics of remifentanil in infants and children during moderate and deep hypothermia to determine how further temperature decreases affect pharmacokinetic parameters. Such future studies should lead to safer more consistently effective dosing of remifentanil in pediatric patients undergoing cardiac surgery with CPB.

## Competing interests

The authors declare that they have no competing interests.

## Authors' contributions

WS performed data analysis and wrote the manuscript. GH and DD designed and performed the study as well as assisted with data analysis and helped write the manuscript. All authors read and approved the final version of the manuscript.

## Pre-publication history

The pre-publication history for this paper can be accessed here:


